# 6-[(2*E*)-3,7-Dimethyl­octa-2,6-dien-1-yl]-5,7-dihy­droxy-8-(2-methyl­butano­yl)-4-phenyl-2*H*-chromen-2-one–6-[(2*E*)-3,7-dimethyl­octa-2,6-dien-1-yl]-5,7-dihy­droxy-8-(3-methyl­butano­yl)-4-phenyl-2*H*-chromen-2-one (1/1) from *Mesua elegans* 1

**DOI:** 10.1107/S1600536812008628

**Published:** 2012-03-03

**Authors:** Gomathi Chan, Khalijah Awang, Nor Hadiani Ismail, Seik Weng Ng, Edward R. T. Tiekink

**Affiliations:** a Department of Chemistry, University of Malaya, 50603 Kuala Lumpur, Malaysia; b Faculty of Applied Sciences, Universiti Teknologi MARA, 40450 Shah Alam, Malaysia; c Chemistry Department, Faculty of, Science, King Abdulaziz University, PO Box 80203 Jeddah, Saudi Arabia

## Abstract

The title co-crystal, C_30_H_34_O_5_·C_30_H_34_O_5_, comprises a 1:1 mixture of two mostly superimposed mol­ecules with the same chemical formula that differ in the nature of the substituent (2-methyl­butanoyl or 3-methyl­butano­yl) bound at the exocyclic ketone. The lactone ring is close to planar (r.m.s. deviation = 0.058 Å) and the phenyl ring is twisted out of this plane [dihedral angle = 60.08 (9)°]. The geranyl substituent is almost normal to benzene ring to which it is connected [C—C—C_ar_—C_ar_ (ar = aromatic) torsion angle = −87.8 (2)°]. Intra­molecular O—H⋯O and O—H⋯π inter­actions are formed. In the crystal, supra­molecular chains are formed along the *a* axis owing to C—H⋯O contacts, with the lactone carbonyl atom accepting two such bonds.

## Related literature

For the spectroscopic characterization of the title material, see: Verotta *et al.* (2004 ▶) and for its acetyl­cholinesterase (AChE) inhibitory properties, see: Awang *et al.* (2010 ▶).
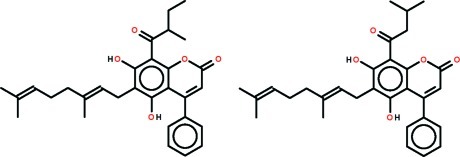


## Experimental

### 

#### Crystal data

C_30_H_34_O_5_·C_30_H_34_O_5_
*M* *_r_* = 949.14
Triclinic, 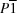

*a* = 5.9426 (2) Å
*b* = 13.4688 (5) Å
*c* = 16.3275 (6) Å
α = 91.955 (3)°
β = 99.515 (3)°
γ = 95.834 (3)°
*V* = 1280.47 (8) Å^3^
*Z* = 1
Cu *K*α radiation
μ = 0.66 mm^−1^
*T* = 100 K
0.30 × 0.15 × 0.05 mm

#### Data collection

Agilent SuperNova Dual diffractometer with an Atlas detector
Absorption correction: multi-scan (*CrysAlis PRO*; Agilent, 2011 ▶) *T*_min_ = 0.826, *T*_max_ = 0.968
25960 measured reflections
5330 independent reflections
4528 reflections with *I* > 2σ(*I*)
*R*_int_ = 0.045

#### Refinement

*R*[*F* ^2^ > 2σ(*F* ^2^)] = 0.066
*wR*(*F* ^2^) = 0.208
*S* = 1.01
5330 reflections
363 parameters
54 restraints
H atoms treated by a mixture of independent and constrained refinement
Δρ_max_ = 0.57 e Å^−3^
Δρ_min_ = −0.53 e Å^−3^

### 

Data collection: *CrysAlis PRO* (Agilent, 2011 ▶); cell refinement: *CrysAlis PRO*; data reduction: *CrysAlis PRO*; program(s) used to solve structure: *SHELXS97* (Sheldrick, 2008 ▶); program(s) used to refine structure: *SHELXL97* (Sheldrick, 2008 ▶); molecular graphics: *ORTEP-3* (Farrugia, 1997 ▶) and *DIAMOND* (Brandenburg, 2006 ▶); software used to prepare material for publication: *publCIF* (Westrip, 2010 ▶).

## Supplementary Material

Crystal structure: contains datablock(s) global, I. DOI: 10.1107/S1600536812008628/hb6652sup1.cif

Structure factors: contains datablock(s) I. DOI: 10.1107/S1600536812008628/hb6652Isup2.hkl

Additional supplementary materials:  crystallographic information; 3D view; checkCIF report

## Figures and Tables

**Table 1:** Hydrogen-bond geometry (Å, °) *Cg*1 is the centroid of the C10–C15 ring.

*D*—H⋯*A*	*D*—H	H⋯*A*	*D*⋯*A*	*D*—H⋯*A*
O4—H4*o*⋯O5	0.85 (1)	1.54 (4)	2.35 (3)	158 (4)
O4—H4*o*⋯O5′	0.85 (1)	1.76 (4)	2.55 (3)	154 (3)
O3—H3*o*⋯*Cg*1	0.84 (2)	2.56 (4)	3.355 (2)	158 (4)
C2—H2⋯O2^i^	0.95	2.47	3.408 (2)	169
C15—H15⋯O2^ii^	0.95	2.59	3.351 (2)	137

Symmetry codes: (i) 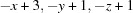; (ii) 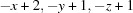.

**Fig. 1.**
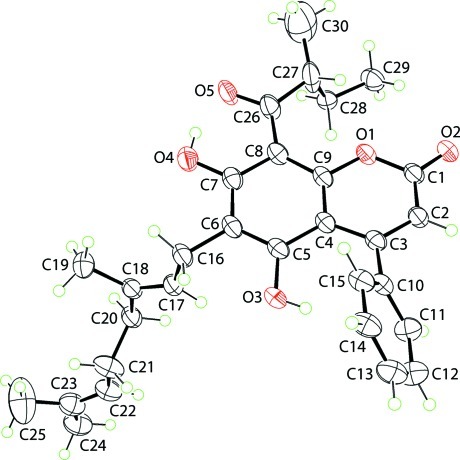
The molecular structure of the first (2-methyl butanoyl) component of (I) showing displacement ellipsoids at the 50% probability level.

**Fig. 2.**
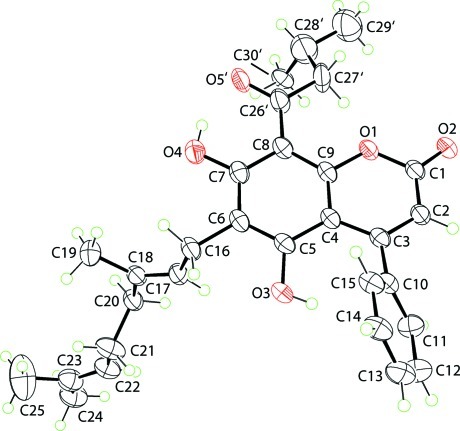
The molecular structure of the second (3-methyl butanoyl) component of (I) showing displacement ellipsoids at the 50% probability level.

**Fig. 3.**
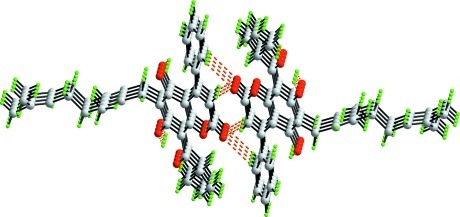
A view in projection down the a axis of the unit-cell contents of (I). The C—H···O interactions are shown as orange dashed lines.

## supplementary crystallographic information

### Comment

The structure of the title co-crystal (I), previously isolated from *Mesua ferrea*, was elucidated by spectroscopic measurements (Verotta *et al.*, 2004). Both components possess the 4-phenylcoumarin skeleton, which is substituted by two hydroxyl group at C-5 and C-7, and a geranyl group at C-6. The difference between the two compounds is the substituent at position C-8; the first is substituted with 2-methylbutanoyl and the second is substituted with the 3-methyl- butanoyl group. The title co-crystal have been evaluated as multi-drug resistant anti-bacterials (Verotta *et al.*, 2004) and for their acetylcholinesterase (AChE) inhibitory properties (Awang *et al.*, 2010). Herein, a crystallographic analysis is described.

The molecular components of the co-crystal (I) are shown in Figs 1 and 2. The r.m.s. deviation for the fitted atoms of the lactone ring = 0.058 Å with maximum deviations of 0.043 (2) Å for the C3 atoms and -0.058 (2) Å for the C4 atom. The phenyl ring is twisted out of this plane, forming a dihedral angle of 60.08 (9)°. The excocyclic carbonyl atom is co-planar with the benzene ring to which it is connected with the C7—C8—C26—O5 torsion angle being 2.2 (10)°; the equivalent torsion angle for the molecule with the 3-methylbutanoyl is 9.9 (9)°. The co-planarity is readily accounted for in terms of intramolecular O—H···O hydrogen bonds. The geranyl group projects almost normal to the plane through the benzene ring with the C5—C6—C16—C17 torsion angle being -87.8 (2)°. The second hydroxyl group forms an intramolecular O—H···π interaction with the phenyl ring, Table 1.

In the crystal, the lactone-carbonyl atom participates in two C—H···O interactions, Table 1, to link molecules into a supramolecular chain along the *a* axis, Fig. 3.

### Experimental

Dried ground bark (1.5 kg) of *Mesua elegans* (*Clusiaceae*), collected from Sungai Badak Forest Reserve, Sintok, Kedah, Malaysia, was extracted with hexane (3 × 4 L, 48 h) at room temperature. The hexane was evaporated to give a yellow gum (120 g). A portion of the extract (10 g) was subjected to column chromatography over silica gel (230–400 mesh) and eluted with hexane:ethyl acetate (from 9.5 to 0) and ethyl acetate:methanol (5:5) to give six fractions. The first fraction was subjected to further silica gel chromatography and eluted with hexane:ethyl acetate (from 9.7 to 9.4) to produce two other sub-fractions. The co-crystal was obtained from the second sub-fraction and recrystallized from its methanol solution as colourless prisms.

### Refinement

Carbon-bound H-atoms were placed in calculated positions [C—H = 0.95 to 1.00 Å, *U*_iso_(H) = 1.2 to 1.5*U*_eq_(C)] and were included in the refinement in the riding model approximation. The hydroxy H-atoms were located in a difference Fourier map, and were refined with a distance restraint of O—H = 0.84±0.01 Å; their *U*_iso_ values were refined.

The crystal is a co-crystal of two molecules having an identical chemical composition. One has an 2-methylbutanoyl substituent in the fused-ring whereas the other has the isomeric 3-methylbutanoyl substituent. As the occupancy refined to a nearly 1:1 ratio, the occupancy of each substituent was set as exactly 0.5.

The pairs of C_benzene_–C_ketone_, C_ketone_–C_methine/methylene_ and C—O distances were restrained to within 0.01 Å of each other, and the anisotropic displacement parameters of the primed atoms were set to those of the unprimed ones. The four-atom unit was restrained to be nearly planar. For the atoms comprising the butyl fragment, 1,2-related C–C distances were restrained to 1.540±0.005 and 1,3-related ones to 2.51±0.01 Å, and the anisotropic displacement parameters were restrained to be nearly isotropic.

### Crystal data

**Table:**

C_30_H_34_O_5_·C_30_H_34_O_5_	*Z* = 1
*M**_r_* = 949.14	*F*(000) = 508
Triclinic, *P*1	*D*_x_ = 1.231 Mg m^−^^3^
Hall symbol: -P 1	Cu *K*α radiation, λ = 1.54184 Å
*a* = 5.9426 (2) Å	Cell parameters from 8956 reflections
*b* = 13.4688 (5) Å	θ = 2.8–76.3°
*c* = 16.3275 (6) Å	µ = 0.66 mm^−^^1^
α = 91.955 (3)°	*T* = 100 K
β = 99.515 (3)°	Prism, colourless
γ = 95.834 (3)°	0.30 × 0.15 × 0.05 mm
*V* = 1280.47 (8) Å^3^	

### Data collection

**Table:**

Agilent SuperNova Dual diffractometer with an Atlas detector	5330 independent reflections
Radiation source: SuperNova (Cu) X-ray Source	4528 reflections with *I* > 2σ(*I*)
Mirror monochromator	*R*_int_ = 0.045
Detector resolution: 10.4041 pixels mm^-1^	θ_max_ = 76.5°, θ_min_ = 2.8°
ω scan	*h* = −7→7
Absorption correction: multi-scan (*CrysAlis PRO*; Agilent, 2011)	*k* = −16→16
*T*_min_ = 0.826, *T*_max_ = 0.968	*l* = −20→20
25960 measured reflections	

### Refinement

**Table:**

Refinement on *F*^2^	Primary atom site location: structure-invariant direct methods
Least-squares matrix: full	Secondary atom site location: difference Fourier map
*R*[*F*^2^ > 2σ(*F*^2^)] = 0.066	Hydrogen site location: inferred from neighbouring sites
*wR*(*F*^2^) = 0.208	H atoms treated by a mixture of independent and constrained refinement
*S* = 1.01	*w* = 1/[σ^2^(*F*_o_^2^) + (0.1357*P*)^2^ + 0.5208*P*] where *P* = (*F*_o_^2^ + 2*F*_c_^2^)/3
5330 reflections	(Δ/σ)_max_ = 0.001
363 parameters	Δρ_max_ = 0.57 e Å^−^^3^
54 restraints	Δρ_min_ = −0.53 e Å^−^^3^

### Fractional atomic coordinates and isotropic or equivalent isotropic displacement parameters (Å^2^)

**Table:**

	*x*	*y*	*z*	*U*_iso_*/*U*_eq_	Occ. (<1)
O1	0.9303 (2)	0.47777 (9)	0.36863 (8)	0.0331 (3)	
O2	1.2727 (2)	0.55978 (10)	0.41312 (9)	0.0390 (3)	
O3	0.7244 (2)	0.13093 (9)	0.41205 (9)	0.0399 (4)	
H3o	0.851 (3)	0.138 (2)	0.4450 (14)	0.052 (7)*	
O4	0.1977 (2)	0.30693 (11)	0.25255 (9)	0.0415 (4)	
H4o	0.198 (6)	0.3641 (14)	0.232 (2)	0.080 (10)*	
O5	0.284 (4)	0.473 (2)	0.218 (3)	0.046 (2)	0.50
O5'	0.308 (4)	0.489 (2)	0.223 (3)	0.046 (2)	0.50
C1	1.1476 (3)	0.48355 (13)	0.41647 (12)	0.0329 (4)	
C2	1.1949 (3)	0.40121 (13)	0.46801 (11)	0.0325 (4)	
H2	1.3363	0.4046	0.5055	0.039*	
C3	1.0417 (3)	0.31880 (13)	0.46420 (11)	0.0311 (4)	
C4	0.8304 (3)	0.30996 (13)	0.40438 (11)	0.0308 (4)	
C5	0.6749 (3)	0.22155 (13)	0.38407 (11)	0.0326 (4)	
C6	0.4647 (3)	0.22110 (13)	0.33394 (11)	0.0340 (4)	
C7	0.4069 (3)	0.31019 (14)	0.29808 (11)	0.0344 (4)	
C8	0.5641 (3)	0.39885 (14)	0.30749 (11)	0.0344 (4)	
C9	0.7747 (3)	0.39437 (13)	0.36031 (11)	0.0314 (4)	
C10	1.0886 (3)	0.24091 (13)	0.52593 (11)	0.0330 (4)	
C11	1.2800 (4)	0.18927 (16)	0.52804 (13)	0.0422 (5)	
H11	1.3861	0.2055	0.4917	0.051*	
C12	1.3170 (4)	0.11372 (18)	0.58338 (15)	0.0508 (6)	
H12	1.4461	0.0774	0.5836	0.061*	
C13	1.1665 (4)	0.09147 (16)	0.63781 (14)	0.0475 (5)	
H13	1.1912	0.0395	0.6751	0.057*	
C14	0.9803 (4)	0.14473 (15)	0.63803 (13)	0.0439 (5)	
H14	0.8794	0.1305	0.6766	0.053*	
C15	0.9392 (4)	0.21920 (14)	0.58222 (13)	0.0390 (4)	
H15	0.8098	0.2552	0.5824	0.047*	
C16	0.3045 (3)	0.12556 (14)	0.31303 (12)	0.0372 (4)	
H16A	0.3228	0.0817	0.3606	0.045*	
H16B	0.1435	0.1414	0.3026	0.045*	
C17	0.3576 (3)	0.07200 (14)	0.23678 (13)	0.0377 (4)	
H17	0.5157	0.0679	0.2355	0.045*	
C18	0.2122 (3)	0.03001 (13)	0.17137 (12)	0.0336 (4)	
C19	−0.0431 (3)	0.02650 (17)	0.16256 (13)	0.0427 (5)	
H19A	−0.0823	0.0682	0.2074	0.064*	
H19B	−0.1095	−0.0426	0.1658	0.064*	
H19C	−0.1049	0.0516	0.1087	0.064*	
C20	0.2995 (3)	−0.01785 (14)	0.09938 (12)	0.0378 (4)	
H20A	0.4624	0.0082	0.1015	0.045*	
H20B	0.2117	0.0022	0.0468	0.045*	
C21	0.2809 (5)	−0.13182 (16)	0.09853 (15)	0.0547 (6)	
H21A	0.3737	−0.1526	0.1499	0.066*	
H21B	0.1191	−0.1584	0.0979	0.066*	
C22	0.3627 (5)	−0.17516 (16)	0.02434 (15)	0.0501 (5)	
H22	0.5179	−0.1552	0.0197	0.060*	
C23	0.2449 (4)	−0.23810 (17)	−0.03575 (14)	0.0501 (5)	
C24	0.3472 (5)	−0.27142 (19)	−0.10876 (16)	0.0590 (6)	
H24A	0.5074	−0.2421	−0.1023	0.089*	
H24B	0.2603	−0.2494	−0.1600	0.089*	
H24C	0.3409	−0.3445	−0.1116	0.089*	
C25	0.0024 (6)	−0.2785 (4)	−0.0393 (2)	0.0989 (14)	
H25A	−0.0533	−0.2536	0.0100	0.148*	
H25B	−0.0096	−0.3517	−0.0407	0.148*	
H25C	−0.0907	−0.2570	−0.0895	0.148*	
C26	0.480 (2)	0.4832 (7)	0.2594 (10)	0.0424 (9)	0.50
C27	0.6207 (16)	0.5821 (7)	0.2569 (5)	0.0590 (13)	0.50
H27	0.7212	0.5942	0.3125	0.071*	0.50
C26'	0.506 (2)	0.4880 (7)	0.2608 (9)	0.0424 (9)	0.50
C27'	0.6741 (18)	0.5785 (8)	0.2554 (6)	0.0590 (13)	0.50
H27A	0.8304	0.5578	0.2586	0.071*	0.50
H27B	0.6755	0.6263	0.3029	0.071*	0.50
C28	0.7818 (6)	0.5736 (2)	0.1937 (2)	0.0311 (7)	0.50
H28A	0.8578	0.5118	0.2025	0.037*	0.50
H28B	0.6900	0.5676	0.1370	0.037*	0.50
C29	0.9641 (7)	0.6620 (3)	0.1995 (3)	0.0458 (9)	0.50
H29A	1.0600	0.6530	0.1570	0.069*	0.50
H29B	1.0598	0.6668	0.2547	0.069*	0.50
H29C	0.8902	0.7235	0.1905	0.069*	0.50
C30	0.4816 (13)	0.6723 (6)	0.2447 (5)	0.117 (3)	0.50
H30A	0.3892	0.6764	0.2889	0.176*	0.50
H30B	0.3803	0.6642	0.1905	0.176*	0.50
H30C	0.5868	0.7336	0.2469	0.176*	0.50
C28'	0.6076 (11)	0.6297 (4)	0.1732 (3)	0.0737 (16)	0.50
H28'	0.4480	0.6489	0.1690	0.088*	0.50
C29'	0.7783 (13)	0.7238 (4)	0.1787 (4)	0.089 (2)	0.50
H29D	0.7365	0.7635	0.1304	0.133*	0.50
H29E	0.9333	0.7046	0.1794	0.133*	0.50
H29F	0.7744	0.7635	0.2298	0.133*	0.50
C30'	0.6254 (7)	0.5651 (3)	0.0980 (2)	0.0362 (8)	0.50
H30D	0.5155	0.5051	0.0944	0.054*	0.50
H30E	0.7814	0.5457	0.1029	0.054*	0.50
H30F	0.5910	0.6025	0.0479	0.054*	0.50

### Atomic displacement parameters (Å^2^)

**Table:**

	*U*^11^	*U*^22^	*U*^33^	*U*^12^	*U*^13^	*U*^23^
O1	0.0368 (7)	0.0244 (6)	0.0359 (7)	−0.0024 (5)	0.0040 (5)	−0.0023 (5)
O2	0.0393 (7)	0.0253 (6)	0.0494 (8)	−0.0036 (5)	0.0038 (6)	−0.0011 (5)
O3	0.0433 (8)	0.0231 (6)	0.0500 (8)	−0.0009 (5)	0.0025 (6)	−0.0037 (5)
O4	0.0383 (7)	0.0393 (8)	0.0431 (8)	−0.0025 (6)	0.0004 (6)	−0.0012 (6)
O5	0.049 (4)	0.029 (7)	0.051 (4)	−0.010 (4)	−0.012 (3)	0.001 (6)
O5'	0.049 (4)	0.029 (7)	0.051 (4)	−0.010 (4)	−0.012 (3)	0.001 (6)
C1	0.0351 (9)	0.0258 (8)	0.0366 (9)	−0.0003 (7)	0.0062 (7)	−0.0049 (7)
C2	0.0335 (9)	0.0274 (9)	0.0351 (9)	−0.0004 (7)	0.0045 (7)	−0.0024 (7)
C3	0.0353 (9)	0.0255 (8)	0.0329 (9)	0.0016 (7)	0.0089 (7)	−0.0044 (6)
C4	0.0338 (9)	0.0259 (8)	0.0323 (8)	−0.0013 (7)	0.0084 (7)	−0.0039 (6)
C5	0.0381 (9)	0.0248 (8)	0.0351 (9)	0.0001 (7)	0.0098 (7)	−0.0042 (7)
C6	0.0366 (9)	0.0285 (9)	0.0359 (9)	−0.0041 (7)	0.0089 (7)	−0.0066 (7)
C7	0.0364 (9)	0.0337 (9)	0.0317 (9)	−0.0013 (7)	0.0061 (7)	−0.0055 (7)
C8	0.0406 (10)	0.0302 (9)	0.0306 (8)	−0.0019 (7)	0.0056 (7)	−0.0032 (7)
C9	0.0355 (9)	0.0263 (8)	0.0315 (8)	−0.0027 (7)	0.0083 (7)	−0.0054 (6)
C10	0.0386 (9)	0.0248 (8)	0.0338 (9)	−0.0015 (7)	0.0052 (7)	−0.0029 (7)
C11	0.0449 (11)	0.0420 (11)	0.0431 (11)	0.0092 (9)	0.0131 (9)	0.0075 (8)
C12	0.0566 (13)	0.0470 (12)	0.0535 (13)	0.0172 (10)	0.0144 (10)	0.0115 (10)
C13	0.0650 (14)	0.0336 (10)	0.0455 (11)	0.0064 (9)	0.0128 (10)	0.0069 (8)
C14	0.0588 (13)	0.0320 (10)	0.0441 (11)	0.0018 (9)	0.0198 (9)	0.0035 (8)
C15	0.0453 (11)	0.0293 (9)	0.0441 (10)	0.0029 (8)	0.0137 (8)	−0.0001 (7)
C16	0.0371 (9)	0.0306 (9)	0.0415 (10)	−0.0047 (7)	0.0067 (8)	−0.0072 (7)
C17	0.0322 (9)	0.0322 (9)	0.0470 (11)	0.0008 (7)	0.0055 (8)	−0.0077 (8)
C18	0.0356 (9)	0.0264 (8)	0.0379 (9)	−0.0008 (7)	0.0060 (7)	−0.0002 (7)
C19	0.0368 (10)	0.0494 (12)	0.0400 (10)	−0.0012 (8)	0.0056 (8)	−0.0044 (8)
C20	0.0386 (10)	0.0322 (9)	0.0409 (10)	−0.0008 (7)	0.0064 (8)	−0.0050 (7)
C21	0.0848 (18)	0.0338 (11)	0.0472 (12)	0.0082 (11)	0.0157 (12)	−0.0017 (9)
C22	0.0640 (14)	0.0358 (11)	0.0521 (13)	0.0076 (10)	0.0146 (11)	−0.0051 (9)
C23	0.0633 (14)	0.0430 (11)	0.0439 (11)	0.0074 (10)	0.0083 (10)	−0.0009 (9)
C24	0.0779 (17)	0.0476 (13)	0.0526 (13)	0.0122 (12)	0.0136 (12)	−0.0113 (10)
C25	0.084 (2)	0.146 (4)	0.0572 (17)	−0.037 (2)	0.0231 (16)	−0.030 (2)
C26	0.048 (3)	0.0361 (12)	0.0364 (11)	−0.0067 (14)	−0.0059 (15)	0.0013 (10)
C27	0.051 (4)	0.0474 (15)	0.0629 (16)	−0.020 (2)	−0.0257 (19)	0.0194 (12)
C26'	0.048 (3)	0.0361 (12)	0.0364 (11)	−0.0067 (14)	−0.0059 (15)	0.0013 (10)
C27'	0.051 (4)	0.0474 (15)	0.0629 (16)	−0.020 (2)	−0.0257 (19)	0.0194 (12)
C28	0.0366 (17)	0.0258 (16)	0.0301 (16)	0.0024 (13)	0.0019 (13)	0.0110 (12)
C29	0.050 (2)	0.0336 (19)	0.054 (2)	−0.0012 (17)	0.0135 (19)	0.0053 (17)
C30	0.115 (6)	0.101 (5)	0.114 (6)	−0.012 (5)	−0.033 (5)	0.032 (5)
C28'	0.078 (4)	0.064 (3)	0.073 (4)	−0.001 (3)	−0.001 (3)	0.005 (3)
C29'	0.101 (5)	0.068 (4)	0.089 (4)	−0.005 (3)	−0.004 (4)	0.017 (3)
C30'	0.051 (2)	0.0264 (16)	0.0316 (17)	−0.0002 (15)	0.0093 (15)	0.0057 (13)

### Geometric parameters (Å, º)

**Table:**

O1—C9	1.368 (2)	C19—H19C	0.9800
O1—C1	1.388 (2)	C20—C21	1.527 (3)
O2—C1	1.213 (2)	C20—H20A	0.9900
O3—C5	1.361 (2)	C20—H20B	0.9900
O3—H3o	0.844 (10)	C21—C22	1.499 (3)
O4—C7	1.335 (2)	C21—H21A	0.9900
O4—H4o	0.853 (10)	C21—H21B	0.9900
O5—C26	1.240 (6)	C22—C23	1.329 (3)
O5'—C26'	1.238 (6)	C22—H22	0.9500
C1—C2	1.437 (3)	C23—C25	1.479 (4)
C2—C3	1.355 (2)	C23—C24	1.500 (3)
C2—H2	0.9500	C24—H24A	0.9800
C3—C4	1.450 (3)	C24—H24B	0.9800
C3—C10	1.494 (2)	C24—H24C	0.9800
C4—C9	1.405 (3)	C25—H25A	0.9800
C4—C5	1.425 (2)	C25—H25B	0.9800
C5—C6	1.375 (3)	C25—H25C	0.9800
C6—C7	1.402 (3)	C26—C27	1.504 (6)
C6—C16	1.514 (2)	C27—C28	1.528 (4)
C7—C8	1.427 (3)	C27—C30	1.538 (5)
C8—C9	1.405 (3)	C27—H27	1.0000
C8—C26	1.483 (6)	C26'—C27'	1.510 (6)
C8—C26'	1.484 (6)	C27'—C28'	1.541 (11)
C10—C11	1.389 (3)	C27'—H27A	0.9900
C10—C15	1.398 (3)	C27'—H27B	0.9900
C11—C12	1.394 (3)	C28—C29	1.516 (4)
C11—H11	0.9500	C28—H28A	0.9900
C12—C13	1.380 (3)	C28—H28B	0.9900
C12—H12	0.9500	C29—H29A	0.9800
C13—C14	1.379 (3)	C29—H29B	0.9800
C13—H13	0.9500	C29—H29C	0.9800
C14—C15	1.391 (3)	C30—H30A	0.9800
C14—H14	0.9500	C30—H30B	0.9800
C15—H15	0.9500	C30—H30C	0.9800
C16—C17	1.511 (3)	C28'—C30'	1.505 (4)
C16—H16A	0.9900	C28'—C29'	1.531 (4)
C16—H16B	0.9900	C28'—H28'	1.0000
C17—C18	1.327 (3)	C29'—H29D	0.9800
C17—H17	0.9500	C29'—H29E	0.9800
C18—C19	1.496 (3)	C29'—H29F	0.9800
C18—C20	1.511 (3)	C30'—H30D	0.9800
C19—H19A	0.9800	C30'—H30E	0.9800
C19—H19B	0.9800	C30'—H30F	0.9800
			
C9—O1—C1	123.73 (14)	C20—C21—H21A	109.3
C5—O3—H3o	108.8 (19)	C22—C21—H21B	109.3
C7—O4—H4o	103 (2)	C20—C21—H21B	109.3
O2—C1—O1	116.26 (17)	H21A—C21—H21B	108.0
O2—C1—C2	127.17 (18)	C23—C22—C21	128.0 (2)
O1—C1—C2	116.46 (15)	C23—C22—H22	116.0
C3—C2—C1	121.27 (17)	C21—C22—H22	116.0
C3—C2—H2	119.4	C22—C23—C25	124.1 (2)
C1—C2—H2	119.4	C22—C23—C24	122.0 (2)
C2—C3—C4	120.15 (17)	C25—C23—C24	113.8 (2)
C2—C3—C10	118.72 (17)	C23—C24—H24A	109.5
C4—C3—C10	121.03 (15)	C23—C24—H24B	109.5
C9—C4—C5	116.29 (17)	H24A—C24—H24B	109.5
C9—C4—C3	118.02 (16)	C23—C24—H24C	109.5
C5—C4—C3	125.66 (17)	H24A—C24—H24C	109.5
O3—C5—C6	114.94 (16)	H24B—C24—H24C	109.5
O3—C5—C4	122.49 (17)	C23—C25—H25A	109.5
C6—C5—C4	122.57 (17)	C23—C25—H25B	109.5
C5—C6—C7	118.48 (16)	H25A—C25—H25B	109.5
C5—C6—C16	121.17 (17)	C23—C25—H25C	109.5
C7—C6—C16	120.24 (17)	H25A—C25—H25C	109.5
O4—C7—C6	116.21 (16)	H25B—C25—H25C	109.5
O4—C7—C8	121.54 (17)	O5—C26—C8	119.2 (19)
C6—C7—C8	122.22 (17)	O5—C26—C27	116.9 (16)
C9—C8—C7	116.14 (17)	C8—C26—C27	123.9 (8)
C9—C8—C26	129.2 (6)	C26—C27—C28	109.4 (9)
C7—C8—C26	114.7 (6)	C26—C27—C30	115.0 (7)
C9—C8—C26'	123.4 (6)	C28—C27—C30	113.0 (5)
C7—C8—C26'	120.5 (6)	C26—C27—H27	106.2
O1—C9—C8	117.12 (16)	C28—C27—H27	106.2
O1—C9—C4	119.40 (16)	C30—C27—H27	106.2
C8—C9—C4	123.48 (16)	O5'—C26'—C8	118.4 (19)
C11—C10—C15	119.28 (18)	O5'—C26'—C27'	117.3 (17)
C11—C10—C3	120.61 (17)	C8—C26'—C27'	124.3 (9)
C15—C10—C3	120.11 (17)	C26'—C27'—C28'	110.6 (7)
C10—C11—C12	120.20 (19)	C26'—C27'—H27A	109.5
C10—C11—H11	119.9	C28'—C27'—H27A	109.5
C12—C11—H11	119.9	C26'—C27'—H27B	109.5
C13—C12—C11	120.2 (2)	C28'—C27'—H27B	109.5
C13—C12—H12	119.9	H27A—C27'—H27B	108.1
C11—C12—H12	119.9	C29—C28—C27	113.2 (5)
C14—C13—C12	120.0 (2)	C29—C28—H28A	108.9
C14—C13—H13	120.0	C27—C28—H28A	108.9
C12—C13—H13	120.0	C29—C28—H28B	108.9
C13—C14—C15	120.48 (19)	C27—C28—H28B	108.9
C13—C14—H14	119.8	H28A—C28—H28B	107.8
C15—C14—H14	119.8	C28—C29—H29A	109.5
C14—C15—C10	119.84 (19)	C28—C29—H29B	109.5
C14—C15—H15	120.1	H29A—C29—H29B	109.5
C10—C15—H15	120.1	C28—C29—H29C	109.5
C17—C16—C6	110.01 (16)	H29A—C29—H29C	109.5
C17—C16—H16A	109.7	H29B—C29—H29C	109.5
C6—C16—H16A	109.7	C27—C30—H30A	109.5
C17—C16—H16B	109.7	C27—C30—H30B	109.5
C6—C16—H16B	109.7	H30A—C30—H30B	109.5
H16A—C16—H16B	108.2	C27—C30—H30C	109.5
C18—C17—C16	128.44 (18)	H30A—C30—H30C	109.5
C18—C17—H17	115.8	H30B—C30—H30C	109.5
C16—C17—H17	115.8	C30'—C28'—C29'	109.5 (4)
C17—C18—C19	123.89 (18)	C30'—C28'—C27'	112.7 (6)
C17—C18—C20	120.64 (18)	C29'—C28'—C27'	105.2 (7)
C19—C18—C20	115.46 (16)	C30'—C28'—H28'	109.8
C18—C19—H19A	109.5	C29'—C28'—H28'	109.8
C18—C19—H19B	109.5	C27'—C28'—H28'	109.8
H19A—C19—H19B	109.5	C28'—C29'—H29D	109.5
C18—C19—H19C	109.5	C28'—C29'—H29E	109.5
H19A—C19—H19C	109.5	H29D—C29'—H29E	109.5
H19B—C19—H19C	109.5	C28'—C29'—H29F	109.5
C18—C20—C21	114.04 (17)	H29D—C29'—H29F	109.5
C18—C20—H20A	108.7	H29E—C29'—H29F	109.5
C21—C20—H20A	108.7	C28'—C30'—H30D	109.5
C18—C20—H20B	108.7	C28'—C30'—H30E	109.5
C21—C20—H20B	108.7	H30D—C30'—H30E	109.5
H20A—C20—H20B	107.6	C28'—C30'—H30F	109.5
C22—C21—C20	111.63 (19)	H30D—C30'—H30F	109.5
C22—C21—H21A	109.3	H30E—C30'—H30F	109.5
			
C9—O1—C1—O2	174.82 (16)	C4—C3—C10—C15	−59.2 (2)
C9—O1—C1—C2	−8.6 (2)	C15—C10—C11—C12	2.7 (3)
O2—C1—C2—C3	−178.28 (18)	C3—C10—C11—C12	−177.2 (2)
O1—C1—C2—C3	5.6 (3)	C10—C11—C12—C13	−1.6 (4)
C1—C2—C3—C4	3.2 (3)	C11—C12—C13—C14	−0.6 (4)
C1—C2—C3—C10	−173.11 (16)	C12—C13—C14—C15	1.7 (3)
C2—C3—C4—C9	−9.5 (3)	C13—C14—C15—C10	−0.6 (3)
C10—C3—C4—C9	166.80 (16)	C11—C10—C15—C14	−1.6 (3)
C2—C3—C4—C5	168.59 (17)	C3—C10—C15—C14	178.26 (18)
C10—C3—C4—C5	−15.1 (3)	C5—C6—C16—C17	−87.8 (2)
C9—C4—C5—O3	169.56 (16)	C7—C6—C16—C17	88.2 (2)
C3—C4—C5—O3	−8.5 (3)	C6—C16—C17—C18	−135.2 (2)
C9—C4—C5—C6	−9.6 (3)	C16—C17—C18—C19	−1.5 (3)
C3—C4—C5—C6	172.29 (17)	C16—C17—C18—C20	178.18 (18)
O3—C5—C6—C7	−175.86 (16)	C17—C18—C20—C21	102.3 (2)
C4—C5—C6—C7	3.4 (3)	C19—C18—C20—C21	−78.0 (2)
O3—C5—C6—C16	0.2 (3)	C18—C20—C21—C22	178.11 (19)
C4—C5—C6—C16	179.46 (16)	C20—C21—C22—C23	−119.9 (3)
C5—C6—C7—O4	−177.50 (16)	C21—C22—C23—C25	−0.6 (5)
C16—C6—C7—O4	6.4 (3)	C21—C22—C23—C24	177.0 (2)
C5—C6—C7—C8	4.2 (3)	C9—C8—C26—O5	−177.9 (19)
C16—C6—C7—C8	−171.91 (17)	C7—C8—C26—O5	2.2 (10)
O4—C7—C8—C9	176.91 (16)	C26'—C8—C26—O5	165 (12)
C6—C7—C8—C9	−4.9 (3)	C9—C8—C26—C27	2.8 (19)
O4—C7—C8—C26	−3.1 (8)	C7—C8—C26—C27	−177.2 (10)
C6—C7—C8—C26	175.1 (8)	C26'—C8—C26—C27	−14 (12)
O4—C7—C8—C26'	−5.2 (8)	O5—C26—C27—C28	−97.7 (14)
C6—C7—C8—C26'	173.0 (8)	C8—C26—C27—C28	81.7 (14)
C1—O1—C9—C8	−177.96 (15)	O5—C26—C27—C30	30.8 (11)
C1—O1—C9—C4	2.4 (2)	C8—C26—C27—C30	−149.8 (11)
C7—C8—C9—O1	178.44 (15)	C9—C8—C26'—O5'	−172.3 (17)
C26—C8—C9—O1	−1.5 (9)	C7—C8—C26'—O5'	9.9 (9)
C26'—C8—C9—O1	0.6 (9)	C26—C8—C26'—O5'	−8 (14)
C7—C8—C9—C4	−2.0 (3)	C9—C8—C26'—C27'	8.4 (18)
C26—C8—C9—C4	178.1 (9)	C7—C8—C26'—C27'	−169.4 (9)
C26'—C8—C9—C4	−179.8 (8)	C26—C8—C26'—C27'	173 (14)
C5—C4—C9—O1	−171.55 (15)	O5'—C26'—C27'—C28'	−27.9 (12)
C3—C4—C9—O1	6.7 (2)	C8—C26'—C27'—C28'	151.4 (12)
C5—C4—C9—C8	8.9 (3)	C26—C27—C28—C29	−167.9 (5)
C3—C4—C9—C8	−172.90 (16)	C30—C27—C28—C29	62.5 (9)
C2—C3—C10—C11	−63.0 (2)	C26'—C27'—C28'—C30'	−65.2 (9)
C4—C3—C10—C11	120.7 (2)	C26'—C27'—C28'—C29'	175.6 (8)
C2—C3—C10—C15	117.1 (2)		

### Hydrogen-bond geometry (Å, º)

Cg1 is the centroid of the C10–C15 ring.

**Table:**

*D*—H···*A*	*D*—H	H···*A*	*D*···*A*	*D*—H···*A*
O4—H4*o*···O5	0.85 (1)	1.54 (4)	2.35 (3)	158 (4)
O4—H4*o*···O5′	0.85 (1)	1.76 (4)	2.55 (3)	154 (3)
O3—H3*o*···*Cg*1	0.84 (2)	2.56 (4)	3.355 (2)	158 (4)
C2—H2···O2^i^	0.95	2.47	3.408 (2)	169
C15—H15···O2^ii^	0.95	2.59	3.351 (2)	137

Symmetry codes: (i) −*x*+3, −*y*+1, −*z*+1; (ii) −*x*+2, −*y*+1, −*z*+1.
